# Upregulation of MAD2L1 mediated by ncRNA axis is associated with poor prognosis and tumor immune infiltration in hepatocellular carcinoma: A review

**DOI:** 10.1097/MD.0000000000032625

**Published:** 2023-01-13

**Authors:** Sizhe Liu, Mingsan Miao, Le Kang

**Affiliations:** a School of Pharmacy, Henan University of Chinese Medicine, Zhengzhou, China.

**Keywords:** hepatocellular carcinoma (HCC), MAD2L1, noncoding RNA (ncRNA), prognosis, tumor immune infiltration

## Abstract

The mortality rate and prognosis of patients with hepatocellular carcinoma (HCC) are well known. A variety of highly malignant human cancers express mitotic arrest deficient 2 like 1 (MAD2L1), a transcription factor that plays a critical role in their development and progression. However, MAD2L1’s particular mechanisms and effects on HCC remain uncertain.

**Methods:**

We performed a pan-cancer analysis for MAD2L1 prognosis and expression using The Cancer Genome Atlas and Genotype-Tissue Expression data in the present study. MAD2L1 may act as an oncogene in HCC, and a combination of in silico analyses, including expression, survival, and correlation analyses, were performed to identify non-coding ribonucleic acids (ncRNAs) that contribute to MAD2L1 overexpression.

**Results:**

In conclusion, MAD2L1 is most likely regulated by HCP5/miRNA-139-5p/MAD2L1 in HCC based on its upstream ncRNA-related pathway. A significant positive association was also found between MAD2L1 levels and tumor immune cell infiltration, immune cell biomarkers, and immune checkpoint expression.

**Conclusion:**

Our findings demonstrate that ncRNA-mediated upregulation of MAD2L1 in HCC is closely related to poor prognosis and tumor infiltration.

## 1. Introduction

Hepatocellular carcinoma (HCC) is one of the most common malignancies in the world. Changes in the social environment and living habits have contributed to the increased incidence and mortality rates in recent years.^[1,2]^ They are a significant threat to human life and health. Currently, HCC is the third most common cause of cancer-related death.^[3]^ Geographical differences and other factors influence HCC tumorigenesis. Most cases occur in underdeveloped regions such as East Asia (54.8% of cases) and Southeast Asia (10.8%).^[3–5]^ Despite considerable advances in diagnosis, treatment, and prognosis, there is still an inferior prognosis for patients with HCC. Therefore, elucidating the molecular mechanisms underlying HCC pathogenesis is very important.^[6]^

Mitotic arrest deficient 2 like 1(MAD2L1) is an essential member of MAD2 family.^[7]^ MAD2L1 is located on human chromosome 4 and is an essential component of the mitotic checkpoint complex protein; disruption of MAD2L1 function in mammalian cells can affect mitotic checkpoint function. It has been found that mutations in MAD2L1 promote tumor development by inducing chromosomal instability and aneuploidy. Multiple types of human cancers, including lung adenocarcinoma,^[8]^ colorectal cancer,^[9]^ cervical cancer,^[10]^ HCC,^[11]^ acute T-cell lymphoma,^[12]^ breast cancer,^[13]^ and stomach cancer^[14]^ are closely associated with MAD2L1.

We used The Cancer Genome Atlas (TCGA) and Gene Expression Profiling Interactive Analysis (GEPIA) databases as part of this review. Analyses have been performed on the expression and survival of MAD2L1 in a wide range of human cancers. The Tumor IMmune Estimation Resource (TIMER) database was used as a next step to investigate the relationship between immune cell infiltration and immune checkpoints in HCC using MAD2L1 expression as a marker. Our results indicated that MAD2L1 is associated with poor prognosis and tumor immune infiltration in patients with HCC.

Our findings suggest that non-coding ribonucleic acids (ncRNAs)-mediated upregulation of MAD2L1 is associated with poor prognosis and tumor immune infiltration in HCC patients. This study provides a theoretical basis for the impact of MAD2L1 on the prognosis of HCC and contributes to the treatment and prognostic survival of patients with HCC.

## 2. Materials and methods

### 2.1. TCGA database analysis

The expression of MAD2L1 in 25 tumor types (glioblastoma multiforme, head and neck squamous cell carcinoma, KICH, kidney renal clear cell carcinoma, KIRP, LAML, LGG, liver HCC, lung adenocarcinoma, lung squamous cell, MESO, OV, PAAD, PCPG, prostate adenocarcinoma, READ, SARC, SKCM, stomach adenocarcinoma, TGCT, THCA, THYM, uterine corpus endometrial carcinoma, UCS, and UVM) was downloaded from the TCGA database (https://genome-cancer.ucsc.edu/). TCGA is a public-funded project that aims to catalogue and discover significant cancer-causing genomic alterations to create a comprehensive “atlas” of cancer genomic profiles.^[15]^ The above data were normalized and analyzed for differential expression of MAD2L1 using the R package limma. Statistical significance was set at *P* < .05.

### 2.2. GEPIA database analysis

Gene expression and cancer profiling were performed using the GEPIA database (http://gepia.cancer-pku.cn/), which is a publicly accessible online resource. It is based on the TCGA and Genotype-Tissue Expression databases^[16]^ used to analyze RNA sequencing data. Fourteen types of tumors were analyzed for overall survival (OS) and RFS survival curves for MAD2L1. Therefore, we evaluated the prognostic value of GEPIA for HCC. Differences were considered statistically significant at a log-rank *P* value of .05. We also evaluated the correlation between MAD2L1 expression and immune checkpoints in HCC, using the GEPIA database. Spearman correlation with default parameters was used to determine coefficients. The statistical significance of this study was determined by an *R* value >0.1 and a *P* value of .05.

### 2.3. Candidate micro RNA (miRNA) prediction

Several target gene prediction programs (microT, miRanda, PicTar, TargetScan, PITA, RNA22, and miRmap) have predicted upstream binding miRNAs of MAD2L1. In subsequent meta-analyses, only predicted miRNAs present in 2 or more procedures were considered. The predicted miRNAs were identified as MAD2L1 candidate miRNAs.

### 2.4. starBase database analysis

The starBase database (http://starbase.sysu.edu.cn/) is used for miRNA target prediction studies. Statistical analysis of miRNA-MAD2L1, long non-coding RNA (lncRNA)-miR-139-5p, or lncRNA-MAD2L1 expression in HCC was performed using starBase. starBase was used to analyze the miR-139-5p expression levels in HCC and normal controls. Additionally, starBase was used to identify potential candidate lncRNAs that could bind to miR-139-5p.

### 2.5. TIMER database analysis

The TIMER database (https://cistrome.shinyapps.io/timer/) is an interactive web application used to analyze tumor-infiltrating immune cells.^[17]^ Based on TIMER, we analyzed the correlation between MAD2L1 expression levels and immune cell infiltration or immune checkpoint expression levels in HCC.

### 2.6. Kaplan–Meier plotter database analysis

The Kaplan–Meier plotter (kmplot.com/analysis) contains data on gene expression and clinical trials. It can be used to analyze the effects of genes or miRNAs on the survival of a variety of cancers. A Kaplan–Meier plot was used to investigate the OS and RFS prognostic values of MAD2L1 messenger RNA (mRNA) and protein expression in HCC.^[18]^ Based on the median gene expression (high vs low), patients were divided into 2 teams for prognostic evaluation. Statistical significance was set at *P* < .05.

## 3. Results

### 3.1. Pan-cancer analysis of MAD2L1 expression

**Figure 1.**
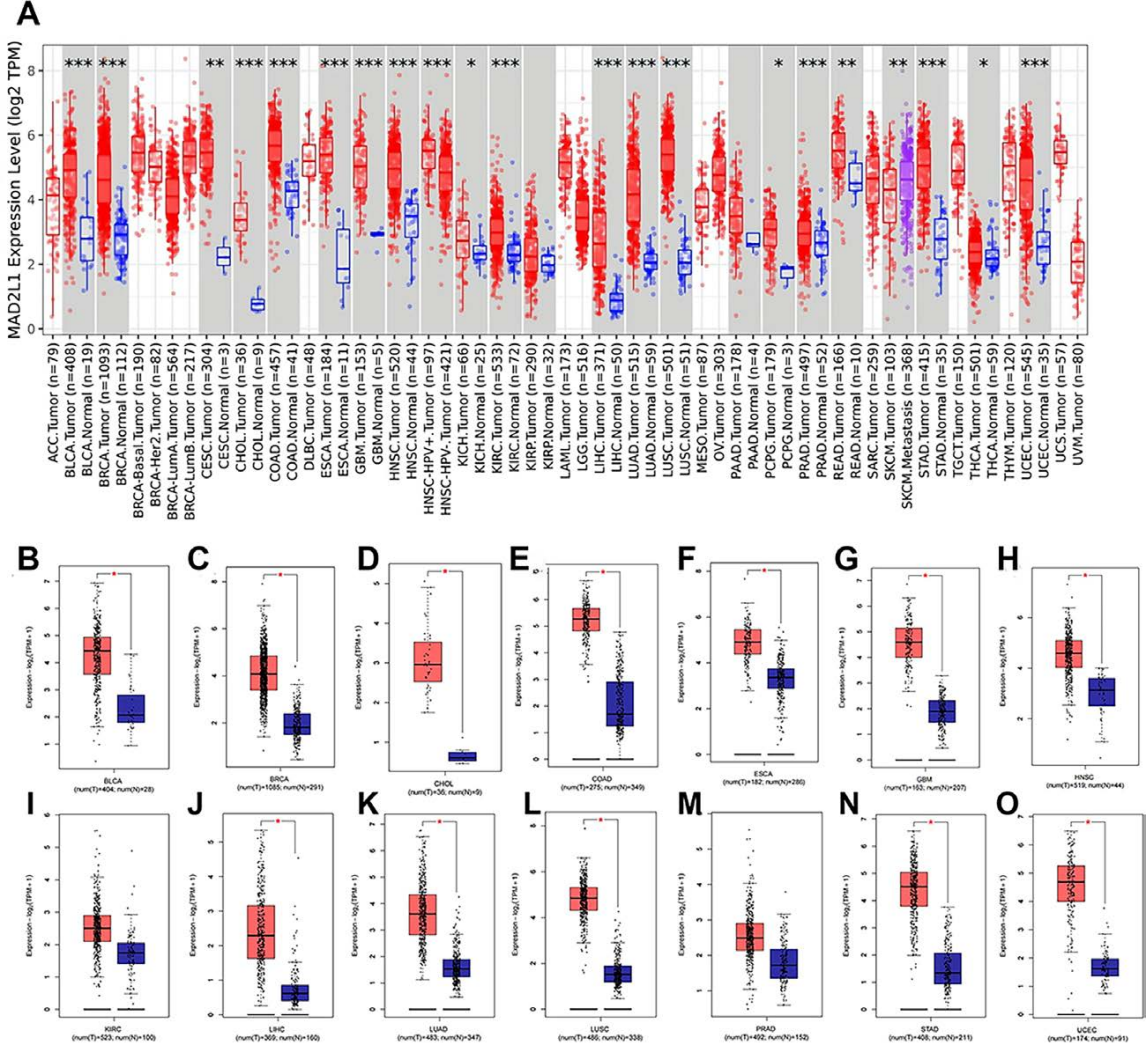
Expression analysis for MAD2L1 in pan-cancers. (A) Using TCGA data from TIMER, we examined the expression of MAD2L1 in different types of human cancers. **P < .01, ***P < .001 (B–O) MAD2L1 expression in (B) TCGA BLCA, (C) BRCA, (D) CHCL, (E) COAD, (F) ESCA, (G) GBM, (H) HNSC, (I) KIRC, (J) LIHC, (K) LUAD, (L) LUSC, (M) PRAD, (N) STAD, and (O) UCEC tissues compared with corresponding TCGA and GTEx normal tissues. *P value <.05; **P value <.01; ***P value <.001. BLCA = bladder urothelial carcinoma, BRCA = breast invasive carcinoma, COAD = colon adenocarcinoma, ESCA = esophageal carcinoma, GBM = glioblastoma multiforme, GTEx = Genotype-Tissue Expression, HNSC = head and neck squamous cell carcinoma, KIRC = kidney renal clear cell carcinoma, LIHC = liver HCC, LUAD = lung adenocarcinoma, LUSC = lung squamous cell, MAD2L1 = mitotic arrest deficient 2 like 1, PRAD = prostate adenocarcinoma, STAD = stomach adenocarcinoma, TCGA = The Cancer Genome Atlas, TIMER = Tumor IMmune Estimation Resource, UCEC = uterine corpus endometrial carcinoma.

As a first step, we analyzed the TIMER2.0 and TCGA databases for MAD2L1 expression levels in all human tumor data. It has been found that MAD2L1 expression in 14 tumor samples is higher than in normal samples, including bladder urothelial carcinoma, breast invasive carcinoma, cholangio carcinoma, colon adenocarcinoma, esophageal carcinoma, glioblastoma multiforme, head and neck squamous cell carcinoma, kidney renal clear cell carcinoma, liver HCC, lung adenocarcinoma, lung squamous cell, prostate adenocarcinoma, stomach adenocarcinoma, and uterine corpus endometrial carcinoma (Fig. 1A). We used the Genotype-Tissue Expression database to validate MAD2L1 expression in 14 cancer types. The expression of MAD2L1 in the above-mentioned tumor tissues was considerably higher than that in normal tissues, as shown in Figure 1B–O.

### 3.2. Prognostic value of MAD2L1 in human cancers

**Figure 2.**
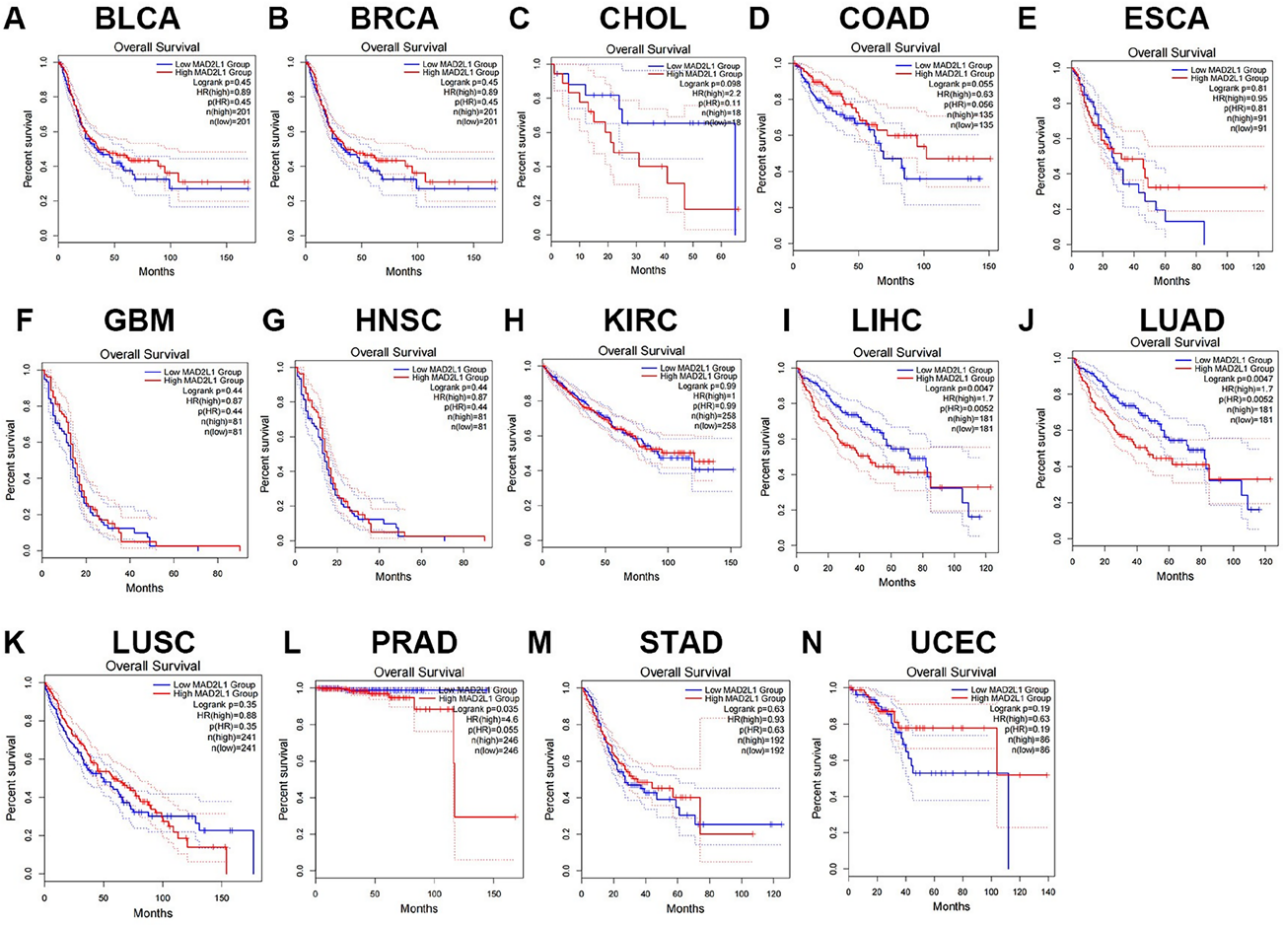
The overall survival (OS) analysis for MAD2L1 in various human cancers determined by the GEPIA database. (A–N) The OS plot of MAD2L1 in (A) BLCA, (B) BRCA, (C) CHOL, (D) COAD, (E) ESCA, (F) GBM, (G) HNSC, (H) KIRC, (I) LIHC, (J) LUAD, (K) LUSC, (L) PRAD, (M) STAD, and (N) UCEC. BLCA = bladder urothelial carcinoma, BRCA = breast invasive carcinoma, COAD = colon adenocarcinoma, CHOL = cholangio carcinoma, ESCA = esophageal carcinoma, GBM = glioblastoma multiforme, GEPIA = Gene Expression Profiling Interactive Analysis, HNSC = head and neck squamous cell carcinoma, KIRC = kidney renal clear cell carcinoma, LIHC = liver HCC, LUAD = lung adenocarcinoma, LUSC = lung squamous cell, MAD2L1 = mitotic arrest deficient 2 like 1, PRAD = prostate adenocarcinoma, STAD = stomach adenocarcinoma, UCEC = uterine corpus endometrial carcinoma.

**Figure 3.**
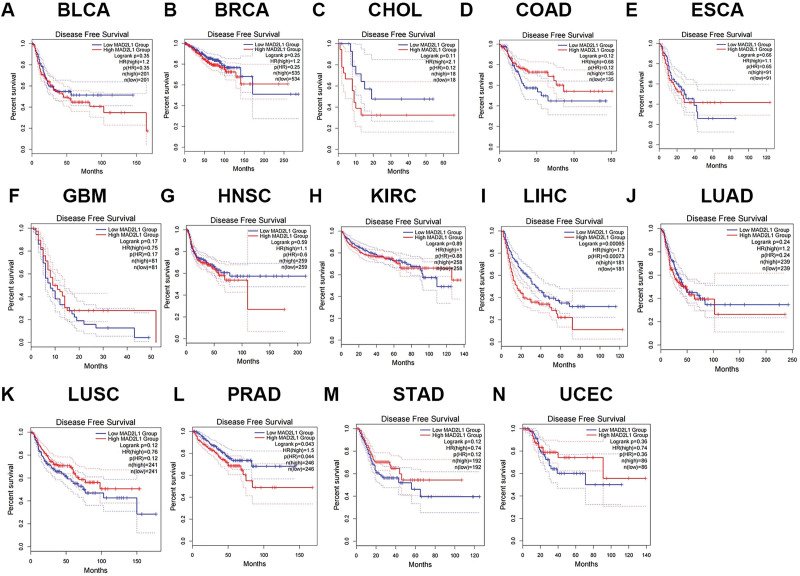
The disease-free survival (DFS) analysis for MAD2L1 in various human cancers determined by the GEPIA database. (A–N) The RFS plot of MAD2L1 in (A) BLCA, (B) BRCA, (C) CHOL, (D) COAD, (E) ESCA, (F) GBM, (G) HNSC, (H) KIRC, (I) LIHC, (J) LUAD, (K) LUSC, (L) PRAD, (M) STAD, and (N) UCEC. BLCA = bladder urothelial carcinoma, BRCA = breast invasive carcinoma, COAD = colon adenocarcinoma, CHOL = cholangio carcinoma, ESCA = esophageal carcinoma, GBM = glioblastoma multiforme, GEPIA = Gene Expression Profiling Interactive Analysis, HNSC = head and neck squamous cell carcinoma, KIRC = kidney renal clear cell carcinoma, LIHC = liver HCC, LUAD = lung adenocarcinoma, LUSC = lung squamous cell, MAD2L1 = mitotic arrest deficient 2 like 1, PRAD = prostate adenocarcinoma, STAD = stomach adenocarcinoma, UCEC = uterine corpus endometrial carcinoma.

Based on the mRNA expression levels of MAD2L1, we divided the cancer cases into high- and low-expression groups. Using Gene Expression Omnibus and TCGA datasets, we examined the association between MAD2L1 expression and prognosis. Two prognostic indices were used: OS and disease-free survival (DFS). Based on these results, MAD2L1 expression has been linked to poor OS and DFS (RFS) prognosis. Analysis of survival curves showed that high MAD2L1 expression in cholangio carcinoma and HCC was associated with poor prognosis for OS. However, patients with colon adenocarcinoma and esophageal carcinoma with higher MAD2L1 expression had better prognosis (Fig. 2). In all cancer types, increased MAD2L1 expression was associated with poor DFS (RFS) in HCC (Fig. 3). By combining OS and DFS, MAD2L1 may be an unfavorable prognostic biomarker in patients with HCC.

### 3.3. Relationship between MAD2L1 expression and prognosis

**Figure 4.**
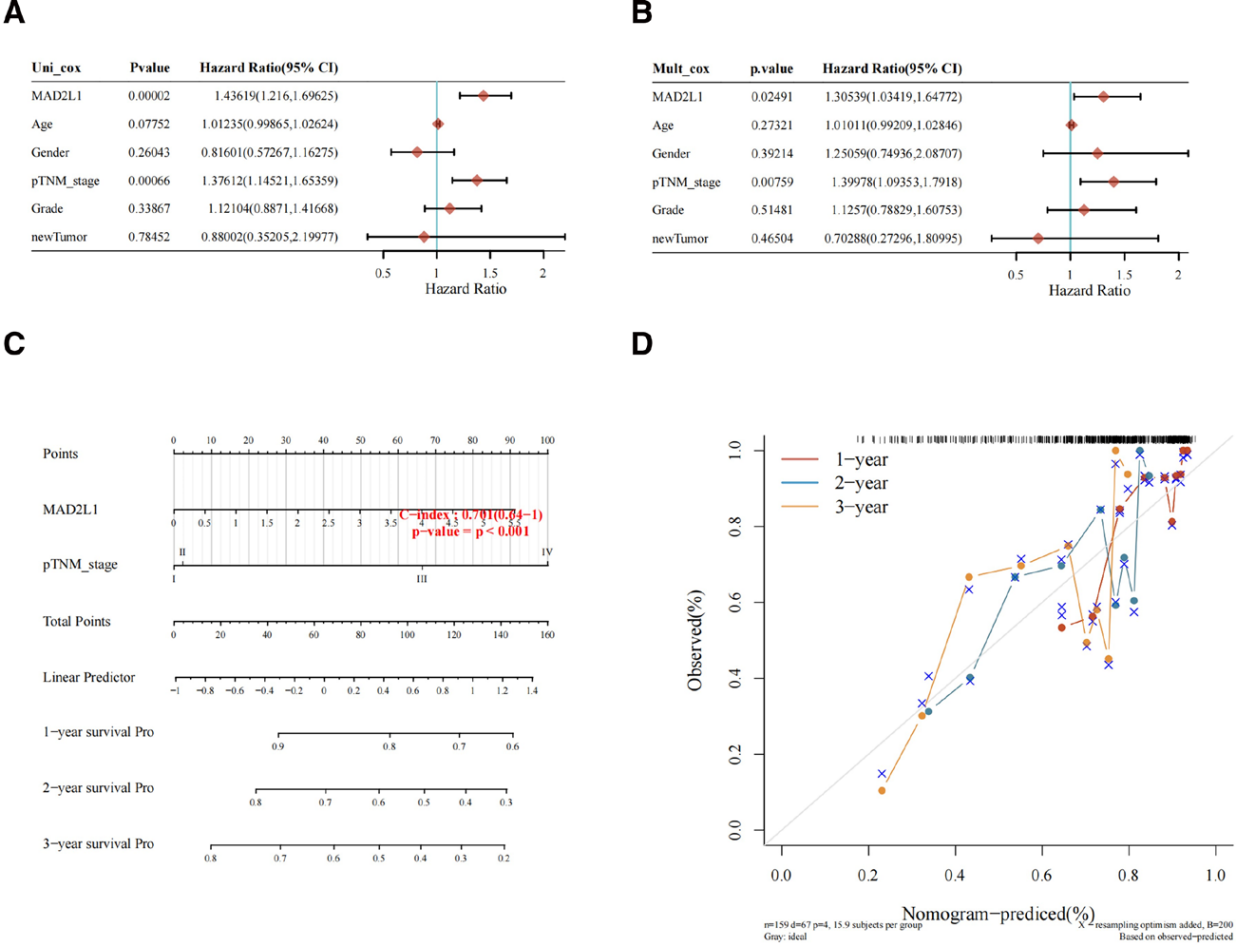
Prognostic prediction model of MAD2L1 in HCC. (A) Univariate Cox analysis of MAD2L1 and other clinicopathological variables. (B) Multivariate Cox analysis of MAD2L1 and other clinicopathological variables. (C) Nomogram for 1-year, 3-year, and 5-year OS in patients with LGG. (D) Calibration plots for 1-year, 3-year, and 5-year OS predictions. HCC = hepatocellular carcinoma, MAD2L1 = mitotic arrest deficient 2 like 1, OS = overall survival.

We next analyzed the association between the risk score, survival time, and MAD2L1 expression profile. Expression (level 3) between downloads from TCGA dataset (https://portal.gdc.com) and the respective clinical information for HCC. Column line graphs were constructed using univariate and multivariate Cox regression analysis. Efforts to map forests were used to display the *P* values, hazard risk, and 95% confidence interval of each variable through the “randomForest” R package. The results suggested that MAD2L1 was an independent prognostic factor, with a C-index of 0.701 (95% confidence interval = 0.64–1) (*P* < .01 (Fig. 4A–C). We developed columnar plots based on multivariate Cox proportional risk analysis to predict overall recurrence rates (1, 3, and 5 years). The column line diagram provides a graphical representation of the factors that can be used to calculate the risk of recurrence for an individual patient through the “RMS” R package by the points associated with each risk factor (Fig. 4D).

### 3.4. Prediction and analysis of upstream miRNAs of MAD2L1

**Figure 5.**
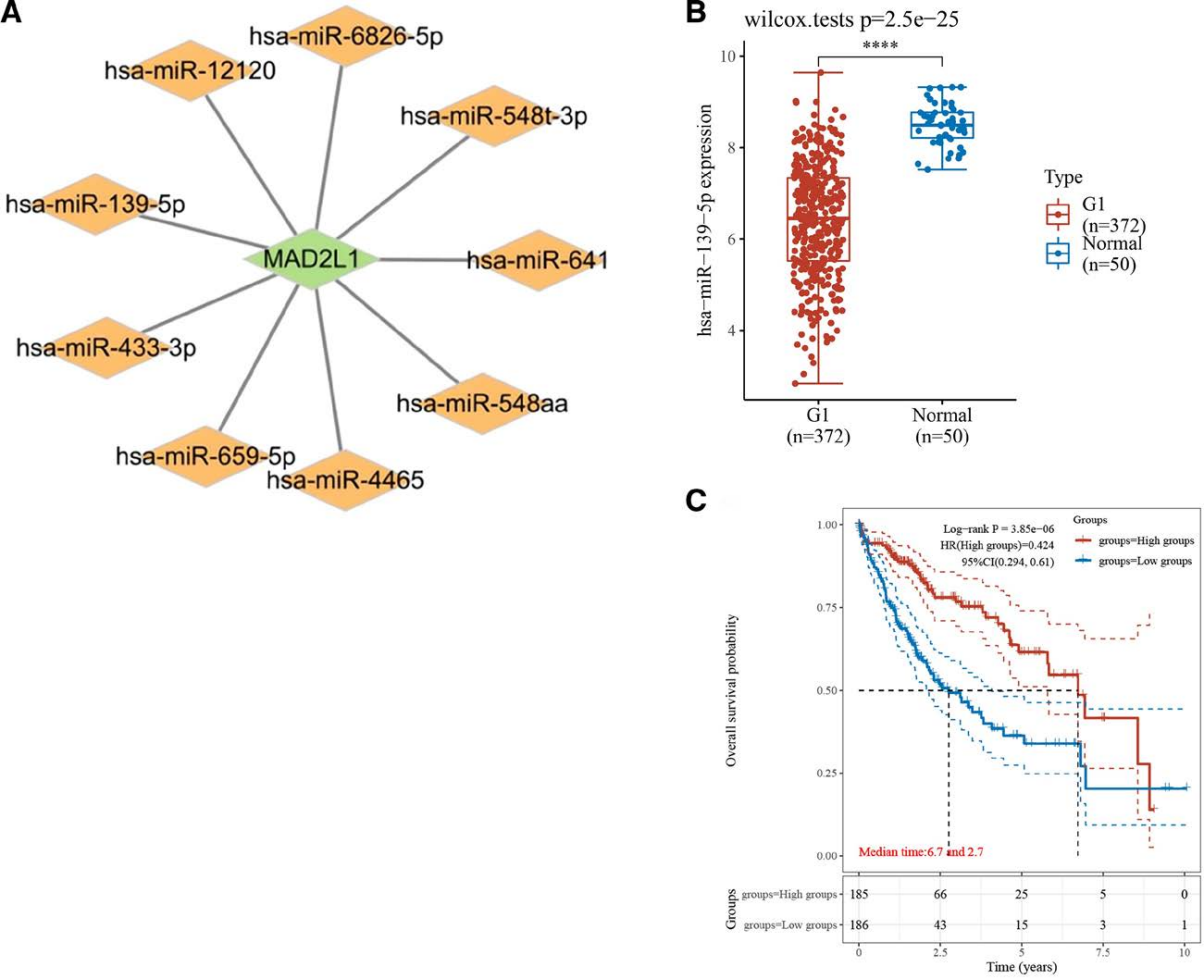
Identification of miR-139-5p as a potential upstream miRNA of MAD2L1 in HCC. (A) The miRNA-MAD2L1 regulatory network was established using the Cytoscape software. (B) The starBase database determines the expression of miR-139-5p in HCC and control samples. (C) Kaplan–Meier plotter was used to assess the prognostic value of miR-139-5p in HCC. HCC = hepatocellular carcinoma, MAD2L1 = mitotic arrest deficient 2 like 1, miRNA = micro RNA.

miRNAs play essential roles in the development of cancer and other diseases. miRNAs target the 3’-UTR of mRNAs to regulate gene expression. The evolution of miRNA expression has been suggested to affect the extent of target regulation, thereby affecting cellular homeostasis. To determine whether miRNAs modulate MAD2L1, we predicted the upstream miRNAs that might bind to the miRNAs and identified 9 miRNAs that bind to it. We used Cytoscape (Fig. 5A) to improve the visualization and establish a regulatory network for miRNA-MAD2L1. The miR-139-5p, miR-433-3p, miR-641, miR-659-5p, miR-548t-3p, miR-548aa, miR-4465, miR-6826-5p, and miR-12120 may bind to MAD2L1. Among the other 8 predicted miRNAs, MAD2L1 showed no significant difference. Finally, we determined the expression and prognostic value of miR-139-5p in HCC patients. miR-139-5p was significantly downregulated in HCC and its upregulation was positively correlated with patient prognosis, as shown in Figure 5B and C. These results demonstrate that miR-139-5p may be the most potentially regulated miRNA in MAD2L1 in HCC.

### 3.5. Prediction and analysis of upstream lncRNAs of miR-139-5p

**Figure 6.**
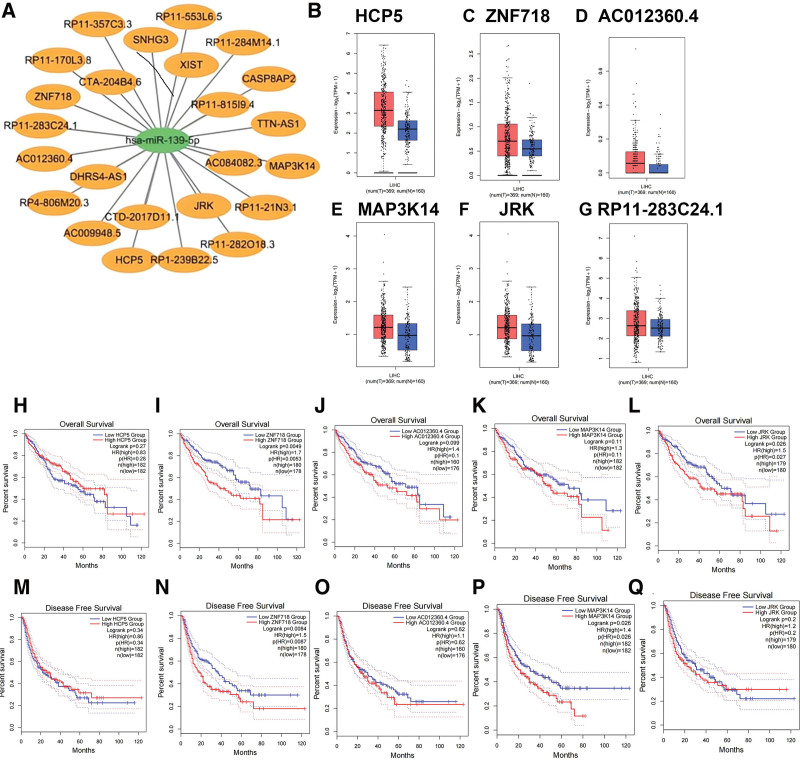
Expression analysis and survival analysis for upstream lncRNAs of miR-139-5p in HCC. The lncRNA-MAD2L1 regulatory network was established using the Cytoscape software. (B–G) Expression of (B) HCP5, (C) ZNF718, (D) AC012360.4, (E) MAP3K14, (F) JRK, and (G) RP11-283C24.1. in TCGA HCC compared with “TCGA normal” or “TCGA and GTEx normal” data. (H–L) OS analysis for (H) HCP5, (I) ZNF718, (J) AC012360.4, (K) MAP3K14, and (L) JRK in HCC. RFS for (M) HCP5, (N) ZNF718, (O) AC012360.4, (P) MAP3K14, and (Q) JRK in HCC. *P value <.05. GTEx = Genotype-Tissue Expression, HCC = hepatocellular carcinoma, lncRNAs = long non-coding RNAs, MAD2L1 = mitotic arrest deficient 2 like 1, OS = overall survival, TCGA = The Cancer Genome Atlas.

The starBase database will be used in the next step to predict miR-139-5p’s upstream lncRNA. A total of 24 lncRNAs were identified. To improve visualization, a miR-139-5p regulatory control network was constructed using Cytoscape (Fig. 6A). We examined the expression levels of these lncRNAs in HCC tissues using the GEPIA database. As shown in Figure 6B–G, compared to normal controls, only HCP5, ZNF718, AC0123460.4, MAP3K14, JRK, and RP11-283C24.1, were significantly upregulated in HCC. Subsequently, the 6 lncRNAs were evaluated for their prognostic value in HCC. Figure 6H–Q shows that only HCC patients with high expression of HCP5 had both poorer OS and RFS; overexpression of JRK indicated poor RFS in patients with HCC. Due to the lack of relevant data, RP11-283C24.1, no OS and RFS analyses were performed. The competing endogenous RNA hypothesis suggests that lncRNAs increase mRNA expression by binding to shared miRNAs. Consequently, there should be a negative correlation between lncRNAs and miRNAs, or a positive correlation between lncRNAs and mRNA. Considering the expression, survival, and correlation analyses, HCP5 might be the most significant upstream lncRNA of the miR-139-5p/MAD2L1 axis in HCC.

### 3.6. MAD2L1 positively correlates with immune cell infiltration in HCC

**Figure 7.**
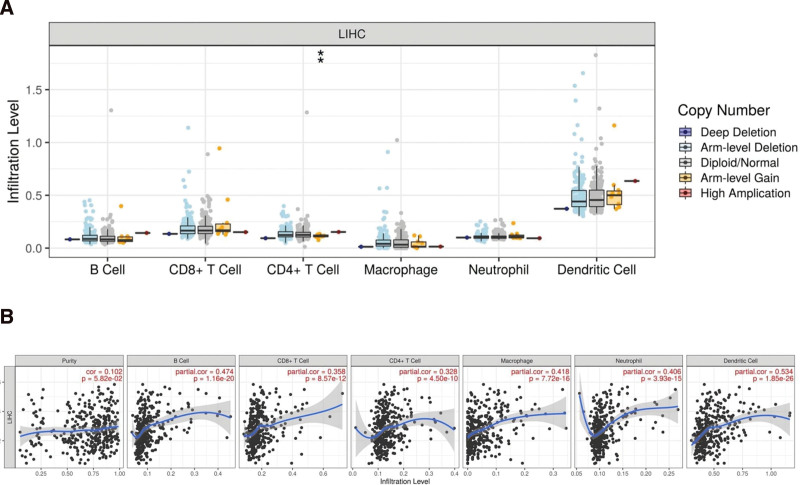
The correlation of MAD2L1 expression level with B cell. (A) Infiltration levels of various immune cells under different copy numbers of MAD2L1 in HCC. (B) Correlation between MAD2L1 expression and B cells. HCC = hepatocellular carcinoma. MAD2L1 = mitotic arrest deficient 2 like 1.

This was followed by an analysis of MAD2L1 immune scores expressed in tumor and normal tissues. Different colors represent different expression distributions according to the samples shown in Figure 7A. *P < .05, **P < .01,***P < .001, asterisks (*) stand for significance levels, and the ordinate represents the expression distribution of immune score in different groups. Correlation analyses can provide critical clues for studying the functions and mechanisms of MAD2L1. We evaluated the correlation between MAD2L expression and immune cell infiltration. As shown in Figure 7B, MAD2L1 expression in HCC was significantly and positively correlated with all immune cells analyzed, including B cells, CD8^+^ T cells, CD4^+^ T cells, macrophages, neutrophils, and dendritic cells. The vertical coordinates represent the distribution of expression in the different groups of the immune score.

### 3.7. Relationship between MAD2L1 and immune checkpoints in HCC

**Figure 8.**
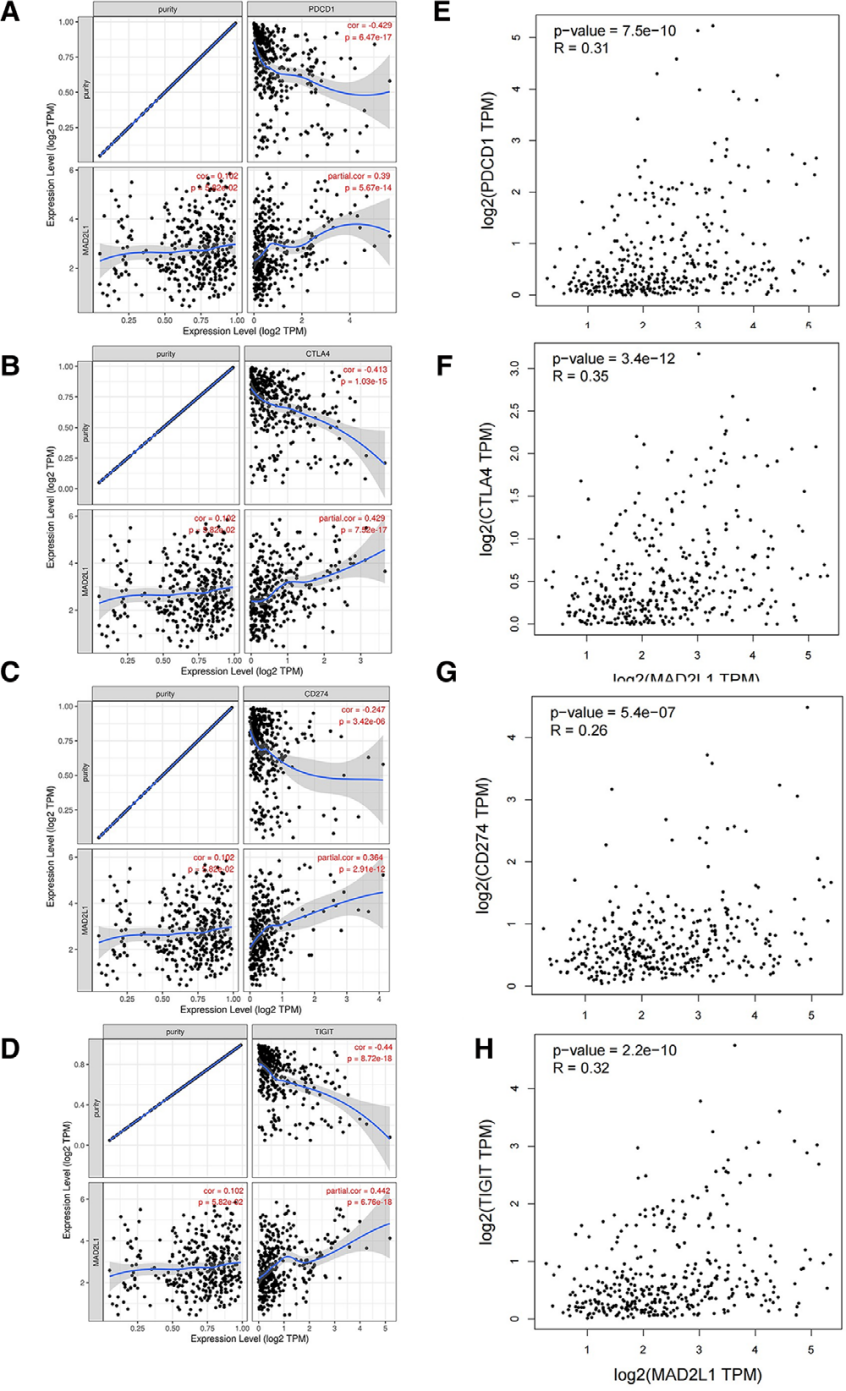
Correlation of MAD2L1 expression with PD-1, CTLA-4, PD-L1, and TIGHT expression in HCC. (A) Spearman correlation of MAD2L1 with the expression of PD-1 in HCC adjusted by purity using TIMER. (B) Spearman correlation of MAD2L1 with an expression of CTLA-4 in HCC adjusted for purity using TIMER. (C) Spearman correlation of MAD2L1 with an expression of PD-L1 in HCC adjusted for purity using TIMER. (D) Spearman correlation of MAD2L1 with TIGIT expression in HCC adjusted for purity using TIMER. (E) The GEPIA database determines the correlation between MAD2L1 and PD1 expression in HCC. (F) The GEPIA database was used to determine the correlation between MAD2L1 and CTLA-4 expression in HCC. (G) The GEPIA database determines the correlation between MAD2L1 and PD-L1 expression in HCC. (H) The GEPIA database was used to determine the correlation between MAD2L1 expression and TIGIT expression in HCC. GEPIA = Gene Expression Profiling Interactive Analysis, HCC = hepatocellular carcinoma. MAD2L1 = mitotic arrest deficient 2 like 1, TIMER = Tumor IMmune Estimation Resource.

The immune checkpoints PD1, PD-L1, CTLA-4, and TIGIT play instrumental roles in tumor immune escape. Considering the potential oncogenic role of MAD2L1 in HCC, the relationships between MAD2L1 and PD1, CTLA-4, PD-L1, and TIGIT were assessed. As shown in Figure 8A–D, MAD2L1 expression in HCC was significantly and positively correlated with PD1, PD-L1, CTLA-4, and TIGIT expression, which was adjusted for purity. Similar to TIMER2.0 data analysis, we also found a significant positive correlation between MAD2L1 expression and PD1, CTLA-4, PD-L1, and TIGIT expression in HCC (Fig. 8E–H). These results suggested that tumor immune escape may be involved in MAD2L1-mediated HCC carcinogenesis.

## 4. Discussion

Owing to its late diagnosis and inadequate treatment options, HCC is becoming an increasing global health problem.^[19]^ Globally, HCC is the fourth leading cause of cancer-related deaths after lung, colorectal, and stomach cancers.^[3]^ By understanding the molecular mechanisms of HCC carcinogenesis, we can develop effective therapeutic targets or search for promising prognostic biomarkers. It has become increasingly clear that MAD2L1 plays a significant role in human cancers, including HCC, during their initiation and progression. Despite this, MAD2L1 remains an unknown factor in HCC that needs to be explored more thoroughly. It has also been reported that HCC is an inflammation-related tumor, where the immunosuppressive microenvironment can induce immune tolerance and evasion through different mechanisms.

In this study, we first conducted a pan-cancer analysis of MAD2L1’s expression using TCGA data, after which the GEPIA database was further employed to validate MAD2L1’s expression. Survival and prognosis analyses for MAD2L1 in these cancer types of interest indicated that HCC patients with high MAD2L1 expression had a poor prognosis. It has been demonstrated that MAD2L1 is associated with deoxyribonucleic acid repair, G2M checkpoint, p53 signaling pathway, PI3K/AKT/mTOR signaling pathway, and Wnt/β-linked protein signaling pathway in HCC.^[11]^ Based on the results of this study, we suggest that MAD2L1 could be used as a potential marker for predicting HCC prognosis.

Previous studies have shown that miRNAs mediate degradation of target mRNAs by binding to 3′-untranslated sequences at the reverse transcription level, thereby inhibiting the expression of target genes.^[20,21]^ To explore MAD2L1’s upstream regulatory miRNAs, we introduced PITA, RNA22, miRmap, microT, miRanda, PicTar, TargetScan, and miRanda prediction programs. Nine miRNAs were identified in this study. These miRNAs exert tumor-suppressive effects on HCC cells. We found that miR-139-5p was markedly downregulated in HCC and its upregulation was positively linked to patient prognosis (*P* < .1). Subsequently, miR-139-5p was selected as the most potent MAD2L1 upstream tumor-suppressive miRNA based on tumor grade expression and survival analyses. Furthermore, previous studies have shown that miR-139-5p suppresses proliferation and migration of liver cancer cells. Wu et al^[22]^ suggested that miR-139-5p decreases the invasion and proliferation capacity of liver cancer tumor cells. In patients with HCC, miR-139-5p expression and sorafenib treatment were not statistically different.^[23]^ Therefore, further studies on the efficacy and expression of miR-139-5p and its associated drugs are warranted.

In recent studies, long noncoding RNAs (lncRNAs) have been linked to cancer progression. Dysregulation of lncRNAs may result in deoxyribonucleic acid, RNA, and proteins that do not play regulatory roles and cause cancer. According to the competing endogenous RNA hypothesis, lncRNAs associated with the miR-139-5p/MAD2L1 axis are potentially oncogenic. We identified 24 potential lncRNAs upstream of the miR-139-5p/MAD2L1 axis. According to expression and survival analysis, HCP5 is the most likely lncRNA to be upregulated in HCC. HCP5 is mainly expressed in immune cells and may affect autoimmunity.^[24]^ Several reports have suggested that HCP5 plays a crucial role in HDGF,^[25]^ AML^[26]^ and other tumors. HCP5 is significantly and highly expressed in HCC tissues compared with normal tissues.^[27]^ rs2244546 in HCP5 is a novel susceptibility locus for HCV-associated HCC development.^[28]^ The HCP5/miR-139-5p/MAD2L1 axis has been identified as a potential regulatory pathway in HCC.

Numerous studies have shown that the interaction between tumor cells and immune cells is essential for cancer development; tumor-infiltrating immune cells affect patient prognosis and antitumor efficacy.^[29]^ This study showed that MAD2L1 is significantly positively correlated with various immune cells, including B cells, CD8^+^ T cells, CD4^+^ T cells, macrophages, neutrophils, and dendritic cells, in HCC. The above studies suggest that tumor immune infiltration may partially explain the mad2l1-mediated oncogenic role in HCC.

Furthermore, the efficacy of immunotherapy requires sufficient immune cells to infiltrate the tumor microenvironment and relies on the adequate expression of immune checkpoints. CTLA4-blocking antibodies and PD1- and PD-L1-blocking antibodies can release anti-tumor immune responses and lead to durable cancer regression.^[30]^ In contrast, ICIs inhibit harmful feedback mechanisms of the immune system by blocking CTLA-4 or PD-1, leading to a durable antitumor response. Therefore, we assessed the relationship between MAD2L1 and immune checkpoints. The results showed that high MAD2L1 expression in HCC was closely associated with PD1, PD-L1, CTLA-4, and TIGIT expression, suggesting that targeting MAD2L1 may improve the efficacy of immunotherapy in HCC.

Altogether, we elucidated that MAD2L1 is highly expressed in multiple types of human cancers (including HCC) and is significantly associated with poor prognosis in patients with HCC. We uncovered a crucial role for MAD2L1 and its upstream regulatory mechanism in HCC, namely HCP5/miR-139-5p/ MAD2L1 axis. Moreover, our results suggest that MAD2L1 may exert oncogenic effects by increasing tumor immune cell infiltration and checkpoint expression. However, these findings should be validated in more basic experiments and extensive clinical trials.

## 5. Conclusions

This study identified a crucial role for MAD2L1 and its upstream regulatory mechanism in HCC, namely HCP5/miR-139-5p/ MAD2L1 axis. It can serve as a new potential prognostic marker for tumors and is positively correlated with immune infiltration in HCC. It provides new evidence for treating HCC and the clinical use of MAD2L1.

## Author contributions

**Conceptualization**: Sizhe Liu, Le Kang.

**Supervision:** Mingsan Miao, Le Kang.

**Visualization:** Sizhe Liu.

**Writing – original draft:** Sizhe Liu.

**Writing – review & editing:** Mingsan Miao, Le Kang.
